# Ultrasonic Nanocrystal Surface Modification: Processes, Characterization, Properties, and Applications

**DOI:** 10.3390/nano12091415

**Published:** 2022-04-20

**Authors:** Akhil Kishore, Merbin John, Alessandro M. Ralls, Subin Antony Jose, Udaya Bhat Kuruveri, Pradeep L. Menezes

**Affiliations:** 1 Quest-Global Engineering Services Pvt Ltd., Bengaluru 560103, India; akhil14247@gmail.com; 2 Department of Mechanical Engineering, University of Nevada, Reno, NV 89557, USA; merbinjohn@nevada.unr.edu (M.J.); alessandroralls@nevada.unr.edu (A.M.R.); subinaj@nevada.unr.edu (S.A.J.); 3 Department of Metallurgical and Materials Engineering, National Institute of Technology, Surathkal, Mangalore 575025, India; udayabhatk@gmail.com

**Keywords:** ultrasonic nanocrystal surface modification, severe plastic deformation, gradient nanostructured layers, microstructure, mechanical properties

## Abstract

Ultrasonic nanocrystal surface modification (UNSM) is a unique, mechanical, impact-based surface severe plastic deformation (S^2^PD) method. This newly developed technique finds diverse applications in the aerospace, automotive, nuclear, biomedical, and chemical industries. The severe plastic deformation (SPD) during UNSM can generate gradient nanostructured surface (GNS) layers with remarkable mechanical properties. This review paper elucidates the current state-of-the-art UNSM technique on a broad range of engineering materials. This review also summarizes the effect of UNSM on different mechanical properties, such as fatigue, wear, and corrosion resistance. Furthermore, the effect of USNM on microstructure development and grain refinement is discussed. Finally, this study explores the applications of the UNSM process.

## 1. Introduction

A dynamic and challenging working environment demands components with superior surface integrity and surface mechanical properties. Enhanced surface integrity and surface mechanical properties play a paramount role in improving the longevity of engineering materials. The literature revealed that more than 80% of mechanical engineering failure, including fatigue, corrosion, and wear, originates from the surface of the components [1]. These failures depend on the surface microstructure and surface properties such as surface hardness, surface roughness, and residual stress state rather than bulk properties [2]. The failure of the mechanical component during their service life brings enormous economic loss. Failures originating from the surface can be prevented by controlling the surface microstructure and the mechanical properties [3,4,5,6]. It is well known that inducing a nanostructured layer on a substrate surface can prevent crack initiation and propagation. The nanostructuring can be conducted either by coating deposition or by adopting different surface modification techniques. However, coating deposition may induce tensile stress between the coating and the substrate, which initiates microcracks. In recent years, surface modification using severe plastic deformation (SPD) techniques or surface nanocrystal (SNC) modification treatment has been widely perceived to improve the surface mechanical properties and surface integrity [7,8]. These treatments induce a nanostructured layer on the surface without affecting the chemical composition of the substrate. These methods can prevent crack initiation and propagation, thereby preventing the onset of the failure. Researchers used various SPD techniques, such as shot peening (SP) [9,10,11], severe shot peening (SSP) [12,13,14], ultrasonic impact peening (UIP) [15,16], ultrasonic shot peening (USP) [17,18,19], laser shock peening (LSP) [20,21,22], direct and indirect laser shock patterning [23,24,25], ultrasonic surface rolling process (USRP) [26,27,28,29], and surface mechanical attrition treatment (SMAT) [30,31,32], to enhance the surface properties. These SPD techniques help to introduce residual compressive stress (RCS) and surface hardening. During the process, gradient nanostructured surface (GNS) layers containing high-density dislocations, twinning, high-angle grain boundaries, and subgrains are formed [33,34]. In the GNS layer, a gradient variation of grain size or lamella thickness is observed. The size varies from the nanoscale on the surface to the microscale in the core of the substrate material [35]. This gradient microstructure can provide the optimum combination of strength and ductility based on the Hall–Petch relationship [36,37]. Surface hardening and nanostructuring during SPD can enhance the wear, fatigue, and corrosion resistance [38].

Even though these SPD techniques improve surface integrity, scholars have searched for techniques that can provide superior control over plastic deformation and surface roughness. In their detailed review, John et al. [8] explained a new SPD technique named “USRP” and its benefit when applied to various engineering materials. They summarized that this method could improve surface mechanical properties without much surface roughness and provide superior control over plastic deformation, which other techniques did not address appropriately. However, a significant increase in surface roughness during multiple passes was an issue observed in USRP. Furthermore, they revealed that the surface hardened during previous rolling passes, and immobile dislocation hindered further plastic deformation and grain refinement. Therefore, scholars put extensive research into developing a method that could accurately control plastic deformation of the treated specimen without much increase in surface roughness. Subsequently, scholars developed another new SPD technique named ultrasonic nanocrystal surface modification (UNSM), which is considered to be a fully controlled metal-dimpling process. The striking density during UNSM is controlled with a computer numerical controller (CNC) to produce a uniform structure with precise control of the surface uniformity [39]. The striking density is such that it produces a micro cold forging which induces severe plastic deformation. Appropriate selection of the process parameters can control the microstructure of the GNS layer, and the surface performance of the substrate can be further improved. In addition to that, the CNC machine tools allow the UNSM process to create an innovative surface on complex geometries without much rise in surface roughness. Furthermore, the mechanical impact can be superimposed with ultrasonic vibration, which allows a precise uniformity in peening. The process parameters can be selected in such a way that the gradient microstructure developed can be accurately controlled, and surface performance can be further improved. The UNSM technique is recommended when superior mechanical properties are the primary concern. The ability of superior control over surface roughness and surface deformation makes it the ideal candidate for strengthening mechanical components when working in extreme and dynamic conditions.

UNSM is a patented technology that uses low-frequency ultrasonic vibrations superimposed on a static load that strikes the substrate surface up to millions of times per second and induces SPD and GNS layer on the substrate surface [40]. UNSM is a high strain rate SPD technique, and the plastic deformation induced due to the mechanical impact improves the substrate’s performance and property. The thickness of the GNS layer depends on the chosen frequency of ultrasonic vibration, static load, and the diameter of the ball, the properties of the ball material, and the substrate [41].

The surface after UNSM treatment has reduced surface roughness and superior mechanical property. UNSM possesses advantages such as ease in operation, low cost, better controllability, and high-efficiency [42]. Scholars have explored many metallic materials whose surface integrity and surface mechanical properties have been improved through the UNSM technique. These are not limited to steel grades [33,34,43,44,45], titanium alloys [46,47,48,49], aluminum alloys [50,51,52], nickel-based superalloys [53,54,55], magnesium alloys [56,57], copper alloys [58,59], shape memory alloys [60,61,62], and high entropy alloys [63,64]. Similarly, it can enhance the surface integrity of the components manufactured through different processing routes, such as additively manufactured (AM) [61,65], sintered [42], and case hardened components [66,67,68]. Researchers demonstrated the effect of UNSM on different materials which reveals the improvement in surface hardness [40,43,57,69,70], introduction of RCS [67,69,70,71,72], enhanced fatigue strength [43,69,71,72], superior grain refinement [43,57,67,73], enhanced wear resistance [40,57,73], improved corrosion resistance [57,70] and reduction in surface roughness [61,74,75].

Numerous reviews on different surface modification techniques are available, ensuring superior mechanical properties. For example, Liu et al. [49] provided a detailed review of the UNSM technique on titanium and titanium alloys. They demonstrated superior tribological properties, enhanced fatigue properties, and improvement in other mechanical properties. However, no review article demonstrates the beneficial nature of UNSM on various engineering materials. Surface modification through UNSM is unexplored and needs to be reviewed comprehensively for various industrial applications. This review paper aims to give a comprehensive overview of the UNSM technique. Section 2 discusses the mechanism of the UNSM. The microstructural development and features of UNSM-treated substrate materials are elucidated in Section 3. The effect of UNSM on mechanical properties is summarized in Section 4. Recent advances are summarized in Section 5. Finally, potential applications of UNSM are explained in Section 6.

## 2. Mechanism of UNSM

**Figure 1:**
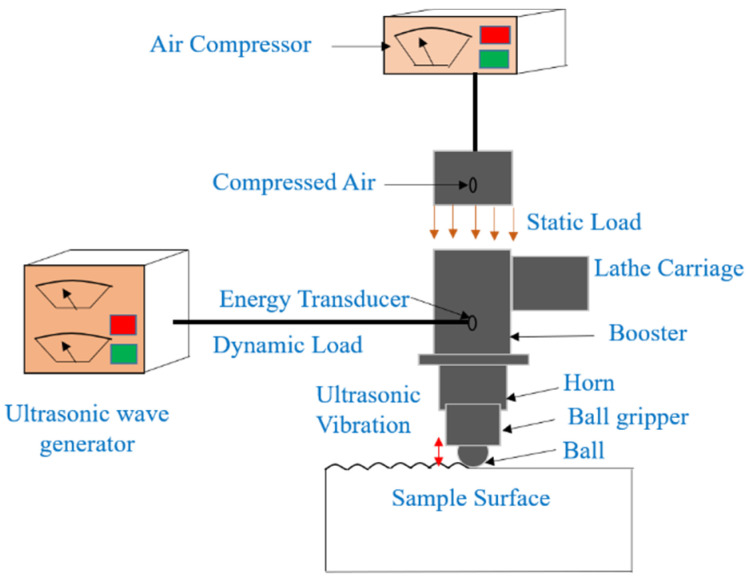
Schematic of UNSM process.

UNSM is presented as an effective, energy-efficient, and economical surface modification technique that can potentially enhance a treated material’s surface integrity and mechanical properties. UNSM is a cold forging technique that induces SPD through the impact of the ball tip on the component’s surface with a very high strike rate. UNSM works on the principle based on transforming a harmonic oscillation into impact energy on the surface imposed with an ultrasonic frequency [76]. A static load is applied to the ball tip and is driven by an ultrasonic transducer which produces tens of thousands of waves per second. The amplitude generated by the ultrasonic generator is in the range of micrometers, which is amplified by an acoustic horn. Hard tungsten carbide (WC) or silicon carbide (SiC) materials are usually used as ball tips. Ultrasonic cold forging technology (UCFT) is another common name used for UNSM. However, the most used name is UNSM. Figure 1 demonstrates the working of UNSM.

A standard UNSM system consists of an ultrasonic wave generator and an air compressor. The static load is produced by compressed air, inducing optimum properties on the specimen surface. UNSM is controlled to perform homogeneous impact strikes and give more accurate control intensity during each sway. Equation (1) shows that the load applied on the surface is the sum of the static and ultrasonic waves [76]. Fs is the static load applied, and Fu is the load added by the ultrasonic waves. Fu changes with processing frequency as ω is 2πf, where f is frequency. Therefore, the load intensity can be changed by changing the Fs component.

(1) Load on the specimen surface=Fs+Fu sin(ωt)

**Figure 2:**
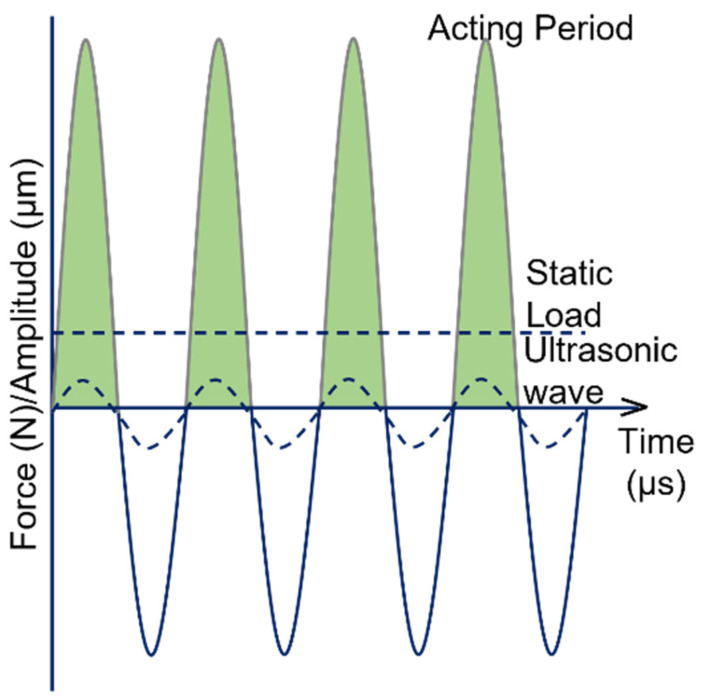
Load cycles in UNSM.

The load wave pattern shown in Figure 2 demonstrates the load acting on the substrate and the force applied. UNSM causes the formation of the nanostructured surface with the GNS layer, demonstrating the synergistic effect of the mechanical impact and ultrasonic vibration. This way, UNSM can provide improved surface mechanical properties and surface integrity.

## 3. Effect of UNSM Treatment on Microstructure Development

UNSM is an SPD technique that can generate a nanocrystalline layer at the material’s surface, leading to significant improvements in mechanical properties, performance level, and useful life of the component [41,45,77]. The extent of formation of the nanocrystalline layer (scale, depth, distribution of nanocrystallites, etc.) depends on the processing parameters and the material being considered [40,45,77,78]. Important processing parameters are load on the specimen surface (which itself has two components, as presented in Equation (1)), vibration strike numbers per unit area, the amplitude of impact, and the diameter of the impacting ball [45,77,78,79]. During UNSM, the energy is transferred from the impacting ball to the material surface. The amount of transferred energy can be compared using strain energy density E as given in Equation (2) [71,79].

E = (FNA)/d(2)

Here, E refers to the energy density available for deformation; also called deformation energy density, it is the amount of elastic and plastic deformation energy transferred to the material surface via the UNSM; F is the load used, N is the number of impacts per area, A is the ultrasonic wave amplitude, and d is the ball tip diameter. In turn, N is obtained using Equation (3)

N = (60f)/(vs)(3)

Here, f is the ultrasonic vibrations, v is the scanning speed of the ball tip, and s is the interval between straight-line paths. In general, an increase in strain energy density (E) increases the amount of strain energy transferred to the surface, hence making it easier to refine the crystal grain. However, the transferred energy dissipates in different ways, such as by loss due to adiabatic heating, defects, and elastic and plastic strain energy. For nanocrystalization, the fraction of plastic strain energy is important, but the fraction itself is a function of the processing variables and material parameters.

High values of plastic strain energy induce SPD in the material. In UNSM, deformation is limited to the material’s surface and hence is also called surface severe plastic deformation (S^2^PD) [45,78]. During each ball’s impact, the deformation is limited to a certain depth from the material’s surface, causing extensive microstructure refinement. The depth and extent of the refinement increase with repeated impact on the surface. The fineness in the microstructure increases, and the lattice becomes distorted due to accumulated micro-strain. Micro-strain in the lattice leads to the formation of the nanostructures. The degree of refinement reduces along the depth direction [40,45,77]. The repeated impact is also responsible for micro-dimples forming on the substrate surface [40]. S^2^PD during UNSM created deformation with strain rates ranging from 0.5/s to 10^6^/s.

**Figure 3:**
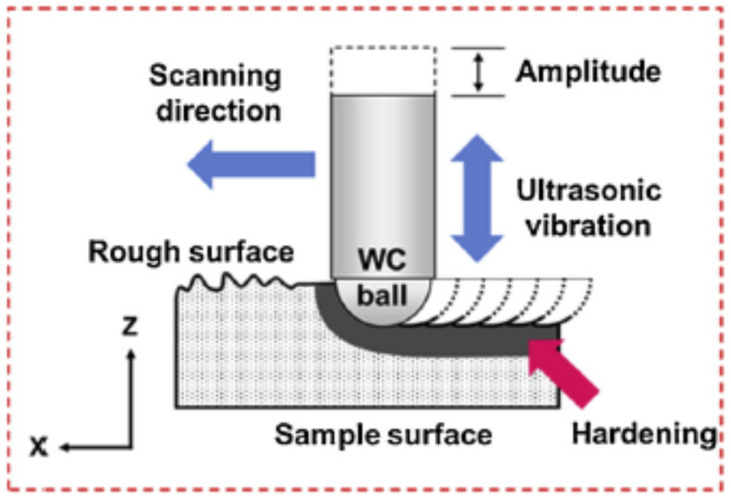
Schematic of generation of a UNSM-modified surface on the material’s surface. Reprinted with permission from ref. [45]. Copyright 2020, Elsevier.

A large strain refines the metal surface by slips, twins, shear bands, etc., creating nanostructures at the surface. It can produce a nanocrystalline surface layer with depths ranging from micrometers to a few tens of micrometers. Gradual transitions in grain size and strain from the surface to the interior lead to a nanostructured material [71]. Using CNC tools, it is possible to position the tool head, precisely leading to a surface that is uniformly modified throughout the surface. Figure 3 presents a schematic of the arrangement producing a modified surface on the material.

Nanocrystallization in different materials

The mechanism of the deformation and refinement of the microstructures during the impact of the UNSM tool head varies from material to material. It is also to be noted that the nanocrystalline surface formation depends on the amount of local strain and the strain rate, temperature, multi-directional deformation, alloying elements, presence of a second phase, etc. [71]. In materials with high stacking fault energy (SFE), such as aluminum and iron, grain refinement involves the formation and rearrangement of dislocations in the form of dense walls and dislocation tangles. Many dislocations are induced in the metallic systems using a high strain rate at low temperatures. When the strain exceeds the threshold value, lattice rotation begins, and the misorientation angle increases, generating nanocrystals. Here, the key to the nanocrystallization of the bulk is to introduce grain boundaries in the surface layers so that its microstructure is transformed into nanocrystallites [30,40,80,81]. For materials such as copper (SFE- 78 mJ/m^2^), dislocation manipulation and rearrangement, deformation twinning forming nanoscale twin-matrix lamellae bundles, and shear banding in the twin/matrix lamellae are the mechanisms of grain refinement [71,82]. In the case of materials such as austenitic stainless steels (ASS), planar dislocation arrays and twins and the intersection of multi-directional twins generate subdivision in the grains. This, along with the strain-induced martensitic transformation, is responsible for the refinement and formation of the nanocrystals. Here, the initial sample would be austenite, and after UNSM, the surface microstructure would be an extremely fine martensite [83,84,85]. In some systems, refinement and nanocrystallization occur by refining the lath martensite and rearranging the martensite units [86].

**Figure 4:**
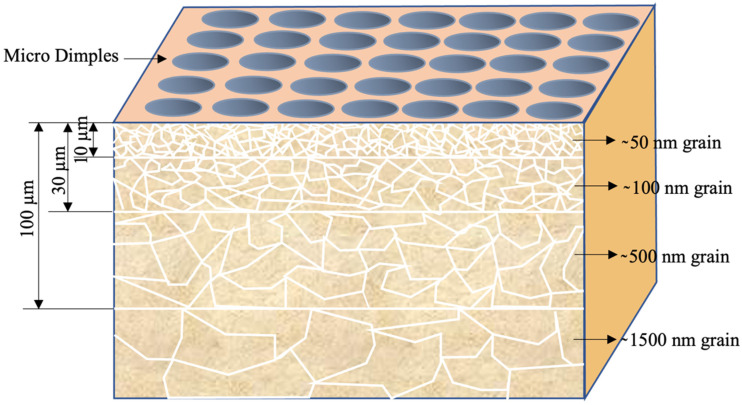
Schematic presentation of the grain refinement on the surface of a material subjected to UNSM. The numbers are only typical ones corresponding to a set of parameters in the selected material.

In the case of double-phase materials, the softer phase is refined first. The presence of a harder phase promotes refinement in the softer phase. Moreover, it is reported that with an increased fraction of the harder phase, the smaller grains are nucleated near the interface between the soft and hard phases due to the easiness of dislocation formation in the soft phase near the hard phase. There are also situations wherein the harder phase is refined first, and then, the dissolution of the second phase particles is reported, as in [87,88,89]. There is another set of materials (such as Mg alloys) where the number of slip systems is limited. Dislocation slip is possible only with selected grains and orientations. Because of the imposed SPD conditions, shear bands are generated within a single grain in multiple directions. Some of the shear bands cross the grain boundaries and continue in nearby grains. In such systems, shear bands initiated at the surface during UNSM propagate to the interior, causing refinement of the grains; the extent is greater at the surface [57]. Whatever the material may be, the UNSM-treated material surface exhibits a top surface that experiences the highest energy effect (most of the time nanocrystallization), followed by regions affected relatively less and finally by unaffected substrate material. Figure 4 represents the gradient microstructure and micro-dimples produced after UNSM.

There are many research investigations on developing nanostructured surfaces using UNSM. The following section elucidates the generation of nanostructures in nonferrous and ferrous-based materials subjected to UNSM.

### 3.1. Nanostructures on the Surface of UNSM-Treated Nonferrous Materials

Oh et al. [52] explored UNSM to develop nanostructures on the surface of Al5083 material. The initial grain size was 132 μm, and after UNSM, it was reduced to an ultrafine size (~1 to 1.5 μm), with Mg-rich precipitates of approximately 50 nm being embedded in the Al-based matrix. The extent of refinement was maximum at the surface, and the effect of UNSM reduced along the depth direction, producing a gradient of nanostructures. Maleki and Teimouri [58] explored obtaining a nanocrystalline copper surface using UNSM. They started with a copper of initial grain size equal to 13 μm and with UNSM were able to achieve 2 μm grains. They proposed that further work with different parameters should be explored to obtain the nanostructured copper surface. Amanov et al. [81] used UNSM to introduce grain refinement at the surface of a Ti alloy, namely Ti6Al4V. Their process parameters generated a deformation depth of about 60 μm. The deformation depth is a function of temperature during processing. The top region deformed at a very high strain rate (10^3^/s to 10^5^/s), leading to extensive grain refinement and producing nanoscaled grains at the surface. Their activity and the dislocation density increased drastically during UNSM. The dislocation movement, dislocation–dislocation interaction, dislocation–grain boundary, dislocation pile-up, etc., increased extensively, contributing further refinement.

**Figure 5:**
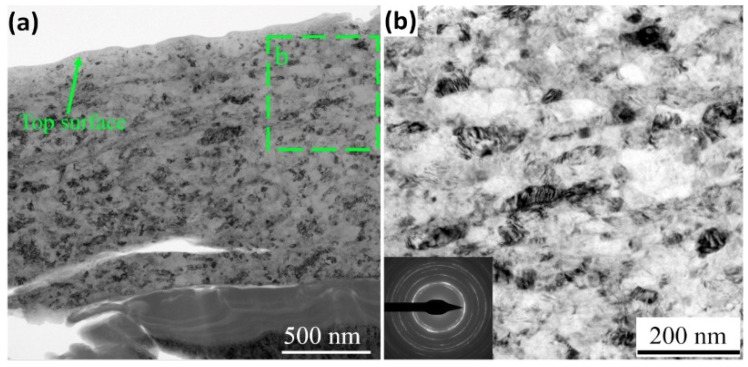
TEM micrograph presenting (**a**) nanostructures on the surface of UNSM-treated Ti6Al4V, (**b**) magnified view of location b. Reprinted with permission from ref. [92]. Copyright 2019, Elsevier.

Furthermore, Zhang et al. [90,91] explored the use of UNSM to improve the fatigue property of 3D-printed Ti6Al4V. The initial microstructure consisted of acicular martensite (α’) morphology. After UNSM treatment, a plastically deformed surface was observed, but deformed martensite could not be differentiated in the scanning electron microscope (SEM). However, in XRD, SPD was observed in the form of peak broadening. The peak broadening was attributed to a reduction in grain size and an increase in dislocation density. Amanov and Pyun [88] subjected Ti6Al4V to UNSM at different temperatures to explore the possibility of nanostructuring at the surface. The X-ray analysis indicated nanostructuring and residual stress at the surface. Liu et al. [92] explored UNSM to bring nanocrystallization to the surface of Ti6Al4V. The initial material had a β matrix in which equiaxed α grains were embedded uniformly. On UNSM, a 10 μm thick layer was deformed. On the top, (1.8 μm depth) nanocrystals of a 25 nm to a 95 nm size were observed. They had irregular boundaries, and in some places, the boundaries were also not clear. These were attributed to deformation during UNSM. Figure 5 presents transmission electron micrographs (TEMs) of nanocrystals on the surface of UNSM-treated Ti6Al4V.

**Figure 6:**
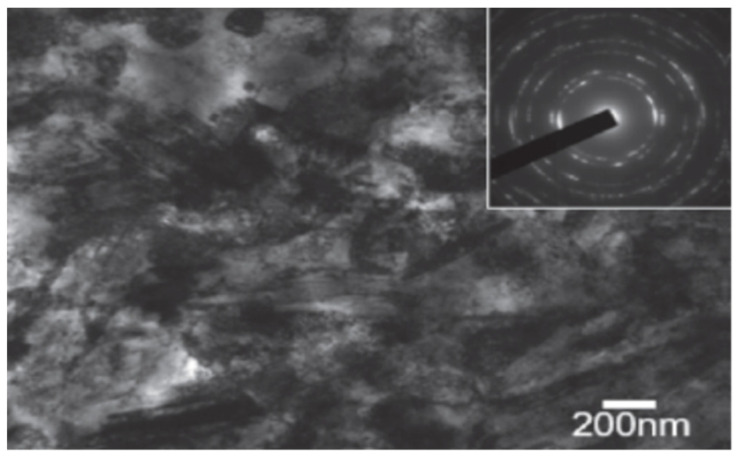
TEM micrograph presenting nanostructured grains developed during UNSM on Inconel 690. Reprinted with permission from ref. [93]. Copyright 2018, Elsevier.

Amanov and Umarov [93] used UNSM to modify the surface microstructure of the Inconel 690 alloy. The untreated material had a nickel-based matrix with an average grain of 32 μm with Cr-rich precipitates along the grain boundaries and TiN inclusions in the matrix and along the grain boundaries. On UNSM, the surface became nanostructured with grains on the scale of 200 nm, as shown in Figure 6. It had extensive mechanical twins with different orientations. Considerable dislocation activity was observed, leading to dislocation accumulation at the grain boundaries, dislocation tangles, and dislocation-assisted sub-boundaries. All these changes led to nanostructuring at the surface, which is a function of the depth from the surface.

Maleki et al. [54] used UNSM to create nanostructures on the surface of the Inconel 718 alloy. They varied the static force in the range of 10 to 50 N by keeping the other parameters constant. The alloy’s initial grain size was about 40 μm, which was reduced to 22 nm (average value measured using X-ray analysis at the surface). The total UNSM affected region was measured at close to 160 μm. Kottoura et al. [69] investigated the effect of UNSM on the Inconel 718 plus alloy. They noted that from an initial grain size of 32 μm (with embedded 30 nm–50 nm sized ϒ’precipitates) the microstructure changed to a nanostructured one at the surface. At the top surface (~1 μm), the crystallites ranged from 10 nm to 50 nm. Below this, the layer had a very high dislocation density with extremely thin deformation twins. In addition, the region presented a cellular structure.

### 3.2. Nanostructures on the Surface of UNSM-Treated Ferrous Materials

**Figure 7:**
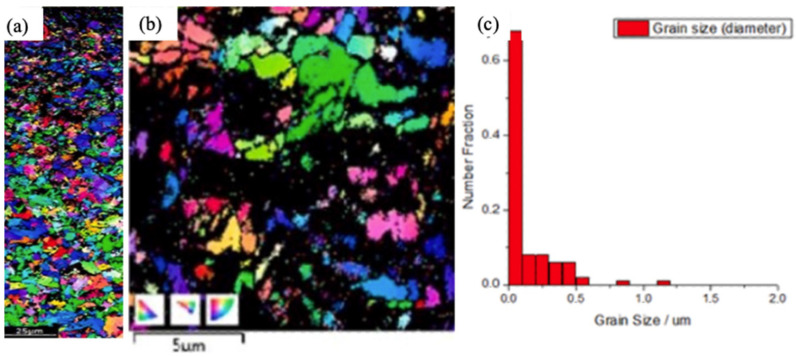
UNSM-treated S500 MC steel substrate: (**a**,**b**) EBSD of the cross-sections at two different magnifications, (**c**) grain-size distribution at the top layer. Reprinted with permission from ref. [97]. Copyright 2021, Elsevier.

The effect of UNSM and the creation of nanocrystallites on the ferrous substrate depends on the type and composition of the substrate. Cao et al. [43] investigated the effect of UNSM on S45C steel. They observed that SPD induces nanograins, and the grain-size gradient develops along with the depth of the steel, without a change in chemical composition. The formation of the nanostructured surface layer from the initial coarse polycrystals involves the generation of dislocations and twinning and the developing of grain boundaries with high-angle misorientation. Using UNSM, from an initial 30 μm grain size, they generated grains of the scale of 50 nm. Though the depth of the nanostructured region was a function of the vibration strike number, the relation was non-linear. By varying the strike numbers from 34,000 times/mm^2^ to 68,000 times/mm^2^, they generated a nanostructured layer thickness of 2 μm to 30 μm. Wu et al. [71] varied static loads in the ranges of 50 N, 60 N, and 70 N to explore the effect of UNSM on S45C steel. The initial material was quenched and tempered with a 20 μm average grain size. Considerably tempered sorbate was observed. After UNSM, they observed a refined layer ranging from 30 μm to 55 μm in depth. X-ray diffraction analysis presented a widening of the diffraction peak, indicating considerable grain refinement. Jo et al. [94] explored the effect of the processing angle during UNSM of S45C steel. They reported a deformation depth of as much as 330 μm. Amanov and Karimbaev [95] explored the effect of UNSM on AISI 4150H steel. Due to water quenching, the base material had a quenched martensite structure, which was refined extensively to generate scale 55 nm–60 nm nanocrystals. Karimbaev et al. [96] treated AISI 4340 steel with UNSM, and they produced a hardened layer of a depth of 275 μm, and at the top, nanograins of 76 nm were observed. Karademir et al. [97] modified the surface of S500 MC automobile steel using UNSM. The initial grain size was 3.7 μm, and after UNSM, they reported an SPD’ed depth of 60 μm, in which the average crystallite size was 100 nm, as shown in Figure 7. The EBSD measurements indicated that more than 85% of the grains were less than 200 nm at the top surface.

Zou et al. [98] modified the surface of DZ2 axle steel using UNSM. They reported three distinct zones in the deformation zone produced due to UNSM. The three zones are a severely deformed topmost surface layer, a transitional layer affected by the shear stress, and an unaffected matrix with equiaxed and coarse grains. They noted that the grains in the topmost layer are compressed and elongated due to static pressure and ultrasonic vibration. The extent of deformation was a function of the number of passes. After the fourth pass, the total deformation depth was 311 μm. Using full width half maximum (FWHM) measurements in X-ray diffraction, they concluded that the top surface had nanocrystallites in the range of 16 nm. Amanov et al. [40] modified the surface of SAE 52100 bearing steel using the UNSM technique. The initial grain size was in the range of 1500 nm. On UNSM, a 100 μm thick modified surface was observed. In this, the top 10 μm thick layer exhibited nanocrystallites on the scale of 50 nm, followed by slightly coarser crystallites of 100 nm (between 10 μm to 30 μm depth). The crystallite size was in the range of 500 nm, in the depth range of 30 μm–100 μm.

**Figure 8:**
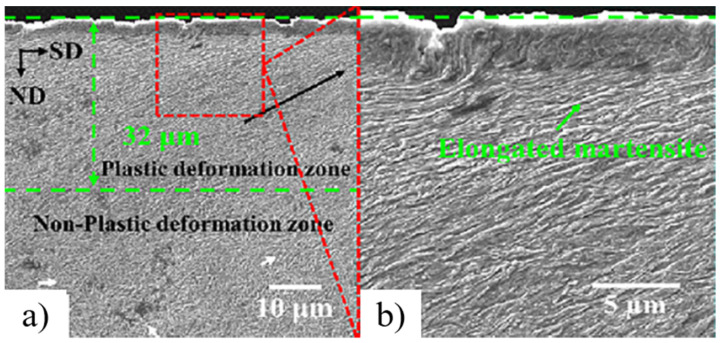
The microstructure of 300 M steel after UNSM treatment: (**a**) the SPD layer, (**b**) the tempered martensite in the SPD layer. Reprinted with permission from ref. [77]. Copyright 2019, Elsevier.

Further down the depth, there was the original grain structure. Zhao et al. [77] subjected 300 M steel to UNSM treatment. The base material was a low alloy, ultra-high-strength steel with tempered martensite as the matrix. S^2^PD using UNSM caused an increase in dislocation density, grain boundaries, and grain refinement. The microstructure was affected up to a depth of 200 μm. The top part (~32 μm) presented refined grains. The top layer presented nanoscale grains (Figure 8). Below the nanoscale grains, the martensite was elongated along the scanning direction and distorted.

Zhao et al. [86] explored the use of UNSM to improve the microstructure of A100 steel to enhance fatigue resistance. The initial martensite was lath martensite with random orientation. After UNSM treatment, the top material up to a depth of about 29 μm was severely deformed. Here, the lath martensite was extensively refined, and the martensite lattice was distorted. The dislocation multiplication and rearrangement were extensive. Even within that, the top 4.1 μm layer was severely deformed, and the martensite was refined greatly. Kim et al. [45] studied the effect of UNSM on AISI M4 tool steel. Before UNSM, the microstructure consisted of austenite and martensite with minor quantities of vanadium carbide, molybdenum carbide, and WC. On UNSM, the austenite was transformed to martensite. This transformed martensite was extremely fine, and they observed more at the surface. This martensite lattice was heavily distorted and strained.

Kim et al. [79] studied the effect of UNSM on AISI D2 tool steel. They explored the role of quench heat treatment given to AISI D2 tool steel on the modification brought by UNSM. The substrate material without heat treatment had a ferritic matrix and a higher strain-hardening capacity. Because of higher ductility, more deformation energy was transferred during the UNSM treatment. The deformation was extended up to 500 μm. Because of the ductility, the extent of deformation was greater, and considerable pile-up took place during each impact. The pile-up was flattened, with re-pile-up during successive pile-ups, increasing the grain refinement. In the case of tool steel, the initial microstructure was martensite with embedded fine carbides with a pre-quench treatment. The martensite was consolidated during each impact of the ball. However, pile-up and strain hardening were not considered due to limited ductility. Excessive coverage leads to microcracks at the bottom of the pile-up material. Eventually, these would peel off as small chips.

**Figure 9:**
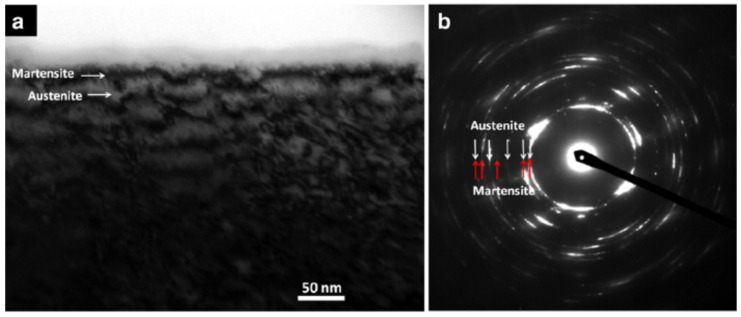
TEM of UNSM-treated AISI 304 steel: (**a**) at the top surface, (**b**) SAED pattern. Reprinted with permission from ref. [44]. Copyright 2013, Elsevier.

There are research works on the nanocrystallization of stainless steel (SS) surfaces using UNSM. Many of these works report the formation of strain-induced martensite on a small scale. Cherif et al. [99] subjected AISI 304 to UNSM treatment. The residual stress analysis revealed that a 0.4 mm depth is affected during UNSM. They reported the formation of strain-induced martensite of a fine scale, up to 35% at the top surface. Moreover, the value of the martensite was more than that obtained from shot peening and deep rolling. Yasuoka et al. [44] reported a gradient in the nanocrystalline plastic deformation layer, up to a depth close to 200 μm. They varied the static load from 70 N to 130 N and observed that the 90 N load gave the highest nanocrystallization. They observed that the surface of the steel was a mix of microstructures of austenite and martensite, as shown in Figure 9. The microstructure scale was several nanometers. At a depth of 20 μm, the scale of the microstructure was a couple of hundred nm. They also reported that SPD associated with 50 microseconds is optimum for rapid martensite transformation. Still, it suppresses the growth of martensite units, and the condition controls the scale of the nanocrystals within several nanometers.

Ye et al. [41] generated a nanostructured layer on the surface of SS304 using UNSM to improve its mechanical properties. They noted that the effect of UNSM is highest on the surface, leading to 100% martensite at the surface. A strain gradient was observed along with the depth, and the martensite fraction was gradually reduced. At a depth of 80 μm, the martensite fraction was 5%. TEM analysis revealed that nanograins of 4.1 nm at the surface and 18 nm at a 10 μm depth were observed. This is an extensive grain-size refinement considering that the initial grain size was 20 μm. Cho et al. [85] subjected AM’ed AISI 316L to UNSM. They noted that more energy density during UNSM leads to increased refinement, producing a gradient in the microstructure from the surface.

## 4. Effect of UNSM Treatment on Fatigue, Wear, and Corrosion Properties

**Figure 10:**
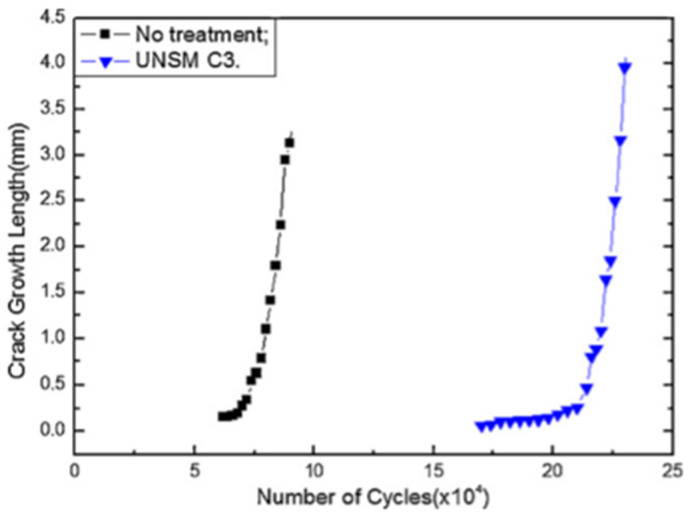
Fatigue crack growth before UNSM treatment and after UNSM treatment. Reprinted with permission from ref. [43]. Copyright 2010, Elsevier.

Surface roughness and surface morphology are two critical parameters that predominantly affect the engineering material’s fatigue, wear, and corrosion properties. Consequently, surface modification techniques are widely employed to prevent failure originating from the surface and to extend the service life. Fatigue performance is an important property that can affect the stability of the components used for structural, automotive, and aerospace applications [100]. Cao et al. [43] performed fatigue experiments on S45C steel subjected to UNSM, with three vibration strike numbers of 34,000, 45,000, and 68,000 times/mm^2^. The specimens corresponding to these vibration strikes were UNSM C1, UNSM C2, and UNSM C3. The authors revealed that with an increase in the vibration strike numbers, the SPD layer thickness increases from 2 µm to 30 µm, and the surface roughness decreases from 1.49 µm to 1 µm. As the vibration strike numbers increase, the fatigue limit is increased. The reduction in the slope of the stress versus the number of cycles to failure (S-N) diagram is a clear depiction of the enhanced fatigue limit. In addition, the authors observed a 33% increase in fatigue strength, corresponding to vibration strikes of 68,000 times/mm^2^, compared to the untreated S45C steel. The authors studied the fatigue fracture surface, and it is presumed that sub-surface fisheye cracks could cause fatigue fracture when the static load is increased. The surface crack growth diagram in Figure 10 validates the presumption that the nanostructured layer delays the crack initiation. The effectiveness of the nanostructured layer in delaying fatigue crack initiation leads to a greater endurance limit of the UNSM C3 substrate. The crack propagation through the nanostructured layer is very slow, and after that, it is very fast and, subsequently, a catastrophic failure. They summarized that UNSM could effectively hinder the propagation of fatigue crack.

Zhang et al. [90] revealed the enhancement in fatigue performance of the 3D-printed Ti64. The UNSM treatment improved the surface finish, reduced the subsurface porosity, and changed the residual stress from tensile to compressive. They considered two UNSM-treated specimens: UNSM-S and UNSM-F, which correspond to the slow and fast rotation of the specimen during UNSM treatment. The authors conducted a rotating bending fatigue (RBF) test, with a rotary speed of 3600 rpm and a stress ratio of −1. The RBF results indicated improvement in fatigue strength for the UNSM-treated specimen for all tested stress levels. The cycles to failure for a stress level of 250 MPa in untreated UNSM-S and UNSM-F were 23,000, 35,000, and 70,000. The RBF on UNSM-F led to a 100% improvement and on UNSM-S led to a 160% enhancement in fatigue strength compared to the base material. The frequency of the ultrasonic vibration is constant in both cases. However, the slower rotary speed in UNSM-S compared to UNSM-F causes a higher number of ultrasonic strikes, which increases SPD, enhances plastic strain, improves surface hardness, and gives a superior surface finish. The authors summarized that UNSM is a potent post-processing method to enhance the fatigue performance of engineering materials.

**Figure 11:**
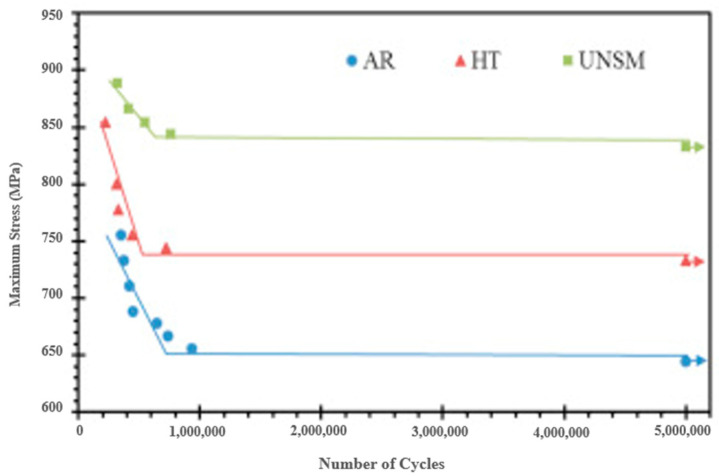
SN curves of AR, HT, and UNSM’ed substrate. Reprinted with permission from ref. [69]. Copyright 2018, Elsevier.

Wu et al. [67] elucidated the effect of using UNSM as a post-treatment to improve the surface integrity of nitrided S45C steel. They performed nitriding for 8 h and 48 h. Even though UNSM treatment improved the fatigue limit of 8 h of nitrided material from 700 MPa to 820 MPa with 34,000 strikes/mm^2^, a decrease in the fatigue limit was observed with an increase in the number of strikes. UNSM treatment on 8 h of nitrided steel improved hardness from 443 Hv to 540 Hv after 34,000 strikes/mm^2^ and 560 Hv after 69,000 strikes/mm^2^. The substrate nitrided for 48 h has shown an even higher hardness of up to 650 Hv. It is inferred that hard particles at the interphase hinder the dislocation movement and act as nucleation sites for dislocations. This phenomenon led to deformation grain refinement in the surface layer, increasing the hardness. More nitriding time implies more nitrogen diffusing into the surface, and more hard particles are formed, further increasing the surface hardness. It is observed that an increase in strike numbers has no significant impact on hardness below 30 µm depth from the surface. Wu et al. [71] conducted UNSM treatment on quenched and tempered S45C steel and reported improvement in the fatigue properties. The untreated specimen possesses a fatigue strength of 464 MPa, whereas the UNSM-treated specimen’s fatigue strength was 523 MPa and 550 MPa. The SPD due to UNSM causes increased surface hardening, which improves the fatigue performance. Kattoura et al. [69] explored the fatigue performance and failure analysis of UNSM’ed nickel-based superalloy (ATI 718 plus). The authors used three specimens in their experiments: as-received (AR), heat-treated (HT), and USNM-treated. UNSM was carried out on the HT specimens. A WC ball with a static load of 40 N and an amplitude of 16 µm was used for the UNSM treatment. Compared to AR, the HT specimens showed a 14% increase in endurance strength. In addition, UNSM improved the endurance strength of the HT specimen at 5,000,000 cycles by 13.5%, which is a 100 MPa increase. The S-N curve for the three specimens is shown in Figure 11. The enhanced fatigue performance of the USNM’ed specimen is attributed to the synergistic effect of surface hardening and RCS.

**Figure 12:**
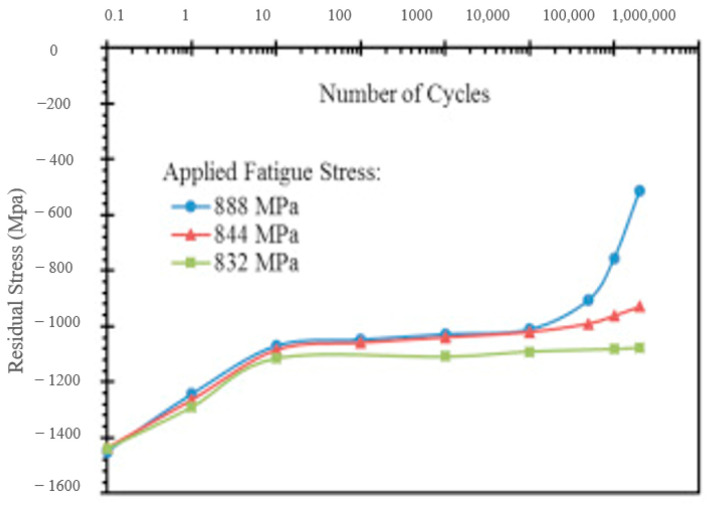
Residual stress relaxation of USNM’ed specimen treated with three fatigue stresses. Reprinted with permission from ref. [69]. Copyright 2018, Elsevier.

A mechanical cyclic relaxation study has been conducted on the UNSM’ed specimen to interpret the effect of the number of cycles on residual stress relaxation during cyclic loading. Three fatigue stresses, such as 888 MPa, 844 MPa, and 832 MPa, were selected for the cyclic relaxation study. At the early stages of cyclic loading, the RCS was suddenly reduced from 1450 MPa to 1100 MPa, which varied slightly with applied stress. A drastic RCS relaxation after 200,000 cycles at the high stress of 888 MPa was observed due to plastic strain, and the specimen failed at 320,000 cycles. This trend was not observed in the intermediate and low stresses, 844 MPa and 832 MPa, respectively, where the fatigue life also increased to 757,000 cycles and 5,000,000 cycles. The residual stress relaxation is shown in Figure 12. These results reveal that the RCS relaxation occurs in the initial cycles and remains constant with slight changes up to 200,000 cycles. The crack propagation rate, calculated from the striation per area, has shown that the UNSM treatment significantly slowed crack propagation by increasing the stress required for micro plasticity. An intact nanocrystalline layer, high dislocation density, subgrains, and thin deformation twins were observed on the fatigue sample. This surface structure significantly contributes to surface integrity and gives a gradient nature of hardness and residual stress distribution [101].

**Figure 13:**
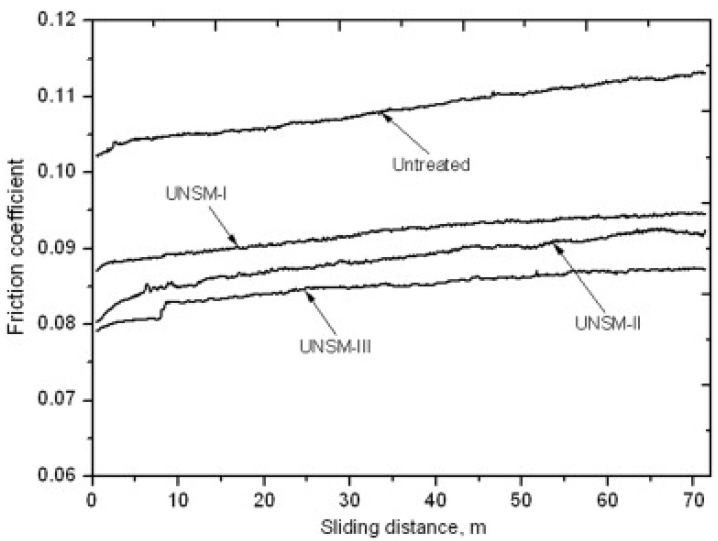
COF variation in untreated and UNSM’ed specimens. Reprinted with permission from ref. [56]. Copyright 2012, Elsevier.

Ye et al. [41] investigated the effect of UNSM on 304 ASS, and their studies revealed an increase in fatigue strength by 100 MPa. The surface hardening during UNSM increased the resistance against the fatigue crack propagation. The improvement in fatigue strength is attributed to the synergistic effect of the RCS, the surface hardening, and the changes in microstructural features due to SPD. Yasuoka et al. [44] explored the effect of UNSM treatment on SUS304 ASS. UNSM treatment is carried out with four static loads, such as 70 N, 90 N, 110 N, and 130 N. The fatigue limit for untreated specimens was 280 MPa. The fatigue strength corresponding to 70 N and 90 N was 430 MPa and 530 MPa. The fatigue strength corresponding to a 110 N and a 130 N static load is lower than other static loads. This is due to the bending of the specimen at a higher fatigue load. The authors summarized that the optimum static load corresponding to UNSM in their experiments is 90 N. Cao et al. [102] has studied fatigue properties of Ti6Al4V shaft, subjected to UNSM treatment with a static load of 25 N, the amplitude of 30 µm, and 36,000 strikes per minute. The authors chose two specimens for UNSM: stress relief annealed (SRA) and solid solution aged (SSA) Ti6Al4V. The coarse polycrystalline grain specimen surface is transformed into a nanostructured one, generating dislocations, twinning, and grain boundaries with high-angle misorientation. The surface microhardness of SRA is 310 Hv, and SSA is 340 Hv. The authors reported that UNSM improves the fatigue strength of SRA substrate by 7% and that of SSA by 11.7%. In UNSM specimens with a fatigue life longer than 10^6^ cycles, a subsurface crack at a depth of 100 µm–200 µm was observed where the hardness and RCS were way below the surface. After UNSM, the microhardness of the SRA specimen increased to 380 Hv (an approx. 22% improvement), and the SSA specimen increased to 395 Hv (an approx. 16% improvement). The researcher perceived a rapid decrease in hardness up to a 120 µm depth from the surface. UNSM on the SRA specimen induced an RCS of 540 MPa, whereas in the SSA specimen, it was 510 MPa. Both UNSM’ed SRA and UNSM’ed SSA showed the same trend in distribution; high compressive stress was observed up to 80 µm from the surface, and a gradual decrease was observed until 200 µm. On a similar note, Suh et al. [103] observed improvement in fatigue strength by 25%, surface hardness by 37%, and RCS by 83% when tool steel SKD-61 was treated with UCFT. The fatigue crack was observed to have originated from the interior in UCFT-treated material, which validates the inhibition of the fatigue crack produced by the nanostructured layer and RCS. On a similar note, scholars revealed the improved wear properties and corrosion of many engineering materials after UNSM treatment. Amanov et al. [56] investigated the effect of UNSM on AZ91D magnesium alloy. They considered three specimens named UNSM 1, UNSM 2, and UNSM 3. These corresponded to a static load of 10 N, 20 N, and 30 N; all the other USNM process parameters remained the same. The friction and wear studies were performed using a ball-on-disk setup with Si_3_N_4_ counter material. The load used for tribological testing varied from 20 N to 100 N. The authors observed an increase in the coefficient of friction (COF) with an increase in sliding distance. Moreover, after 45 m of sliding, the COF was almost stable for the UNSM’ed specimen. This is attributed to the presence of dimples on the surface due to UNSM. These micro-dimples were bulges worn out, providing easy sliding after completing a certain sliding distance. The COF for the untreated specimens was higher than the UNSM’ed specimens. Figure 13 demonstrates the effect of UNSM on COF. It is noted that microhardness was improved from 230 Hv to 295 Hv along with enhancements in the tribological properties. This study showed a marginal increase in hardness with an increase in static load, which became insignificant at a depth of 100 µm. Hindrance to dislocation movement caused by the grain boundaries and dislocation multiplication due to dislocation pile-up are the main factors influencing the hardness of the material. However, the Hall–Petch relation may not hold well if the grain size is less than 10 nm. Table 1 demonstrates the changes in mechanical properties and microstructural changes in the UNSM.

**Figure 14:**
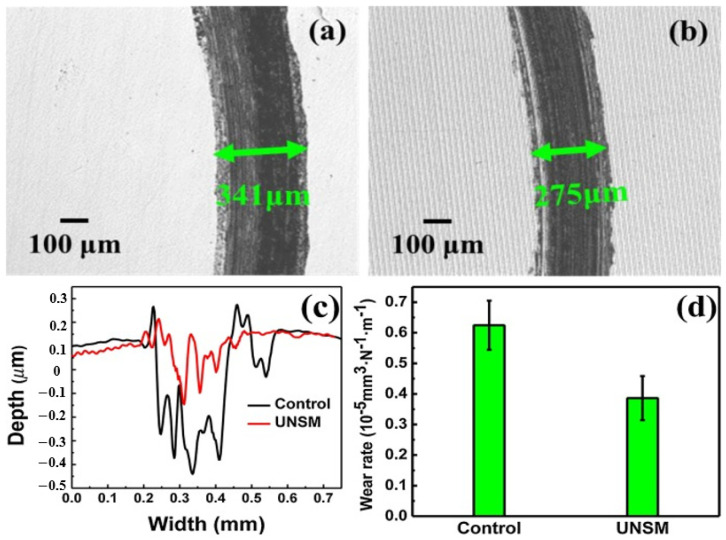
The wear track profiles of the (**a**) bulk and (**b**) UNSM 300 M martensitic ultra-high-strength steel, (**c**) wear depths, and (**d**) wear rates. Reprinted with permission from ref. [73]. Copyright 2020, Elsevier.

Scholars reported that UNSM could provide superior tribological and corrosion properties. Zhao et al. [73] also studied the effects of UNSM on the tribological performance of 300 M martensitic ultra-high-strength steel. In this work, a WC tool of 2.38 mm diameter was applied at a 20 kHz frequency along the surface of the 300 M steel. Through varying the loads (30 N, 40 N, and 50 N) and scanning speeds (250 mm/min, 500 mm/min, and 1000 mm/min), the primary goal of this work was to determine the influence of UNSM on a material that has a body-centered tetragonal (BCT) martensitic structure. The findings of this work conspicuously showed that the processing condition of a 250 mm/min scanning speed and 50 N load resulted in nearly a 40% decrease in wear rate compared to the original substrate, as shown in Figure 14.

According to Zhao et al. [73], the intrinsic enhancements influenced by UNSM altered the wear mechanisms of the steel substrates. For the non-processed sample, it was observed that there was severe abrasion along the wear track, whereas UNSM changed the wear mechanisms to a more adhesive, fatigue, and oxidative wear mode. This was made especially evident by the higher concentrated carbon, oxygen, and tungsten content along the wear track, thus reflecting that the formation of a protective oxide film on the surface was present during triboloading. As reported by Zhao et al. [73], three core mechanisms enabled these findings. First, the accumulation and entanglements of dislocations within the subgrain boundaries of the martensitic structure (from the high plastic strain of the UNSM process) allowed the surface to become increasingly work-hardened. By forming various twins, solid solution strengthening occurred due to the number of supersaturated carbon atoms in the structure, allowing the surface to better resist the tribological loads. Second, the severe strain induced by UNSM healed the pre-existing surface defects (i.e., micro-cracks), as reflected by the relatively low surface roughness. In fact, the presence of micro-cracks (whether they are already intrinsic or induced from porous surfaces) can result in an early brittle fracture as the stress concentrations from the applied triboload result in the emission of localized dislocations [107]. Consequentially, third body wear can occur from the accumulated debris (from fractured asperities to larger macro-sized debris), increasing the friction and wear rates [108,109,110]. Lastly, the large degree of RCS improved the quality of the surface, which similarly maintained the working integrity of the surface (via inhibiting the degree of micro-cracks) as it was subjected to triboloading. By combining these aspects, the wear rate of UNSM 300 M martensitic ultra-high-strength steel was reduced.

Amanov et al. [111] also observed a notable decrease in wear rate from UNSM. In their work, a WC-Co coating on a heat-treated SAE 52100 bearing steel substrate was fabricated through a high-velocity oxygen fuel (HVOF) process. Post-deposition, the as-sprayed surface was subjected to the UNSM process. The applied parameters consisted of a 20 kHz frequency, 10 N static load, 30 μm amplitude, 0.07 mm interval, and 2.38 mm ball diameter (being composed of WC). Once processed, the coatings were subjected to a ball-on-disk tribotest. The sliding distance was set to 10 mm, with the load progressing from 20–120 N while keeping a constant sliding speed of 20 mm/min. Similar to the earlier discussions, the dominant grain-refining mechanism of UNSM enabled a higher degree of surface hardening, which consequentially decreased the overall wear rate. However, the novelty which the work presents is through the relationship of the wear rate to the change in surface roughness and the adhesive/cohesive coating strength.

**Figure 15:**
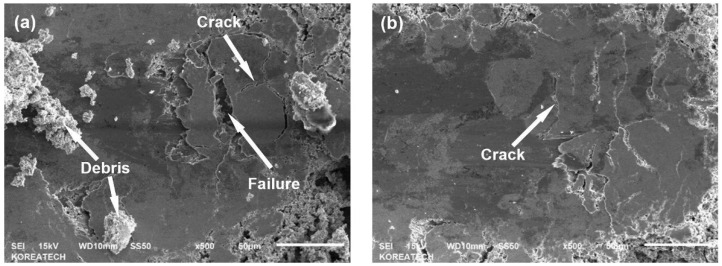
The surface morphology of the (**a**) base and (**b**) USNM’ed HVOF SAE 52100 bearing steel. Reprinted with permission from ref. [111]. Copyright 2021, Elsevier.

Given that the surface roughness (*R_a_*), skewness (*R_sk_*), and kurtosis (*R_ku_*) values of the as-fabricated coating were high, the intense peening effects of the UNSM process resulted in a decrease of these surface parameters. From a tribological perspective, this is significant as the reduction in peaks (influenced by *R_sk_*) and the blunting of asperities (influenced by *R_ku_*) can reduce the stress concentrations along the surface during triboloading. However, in the case of high-stress concentrations, the likelihood of third-body wear again increases, which can negatively influence the wear rates. In fact, there was also a decrease in small-sized pores throughout the surface as the localized valleys were filled and densified, which also assisted with the improved wear resistance. From another perspective, this densification also improved the particle-to-particle bonding of the peened region, which prevents particle debonding and fracturing during triboloading. Amanov et al. [111] support this observation by calculating the difference in adhesion energy of the tested specimens, which was calculated using the following equations:

(4) $$\begin{array}{c} W=K_{1}{\left(\sigma_{s}+\sigma_{R}\right)}^{2}t\frac{1-v_{f}^{2}}{E_{f}}\;\;\;\;\;\;\;\;\;\;\;\;\;\;\;\;\;\;\;\;\;\;\;\; \end{array}$$

(5) $$\begin{array}{c} \sigma_{s}=\frac{0.15}{R}{\left(\frac{PH_{f}}{H}\right)}^{0.5}E_{f}^{0.3}E^{0.2}\;\;\;\;\;\;\;\;\;\;\;\;\;\;\;\;\;\;\;\;\; \end{array}$$

where W is the adhesion energy,$\;K_{1}$ is the corresponding spallation constant, $\sigma_{s}$ is the stress induced from the scratch test, $\sigma_{R}$ is the residual stress, $v_{f}$ is Poisson’s ratio, $E_{f}$ is the coatings elastic modulus, *R* is the radius of the indenter, P is the critical load, H is the hardness of the substrate, $H_{f}$ is the hardness of the coating, and E is the coatings elastic modulus. Considering these values, the adhesive energy increased from 42.4 J/m^2^ to an improved value of 71.3 J/m^2^, which was evident from the reduction in initiated cracks along the wear track (as shown in Figure 15).

When the wear track was characterized through EDX mapping, the non-processed HVOF specimen depicted a high concentration of Fe within the wear track, indicating an adhesive failure within the coating. Contrary to these findings, there was only a partial inception of Fe detection for the peened specimen, thus further validating the effectiveness of UNSM for not only bulk components but also coatings.

**Figure 16:**
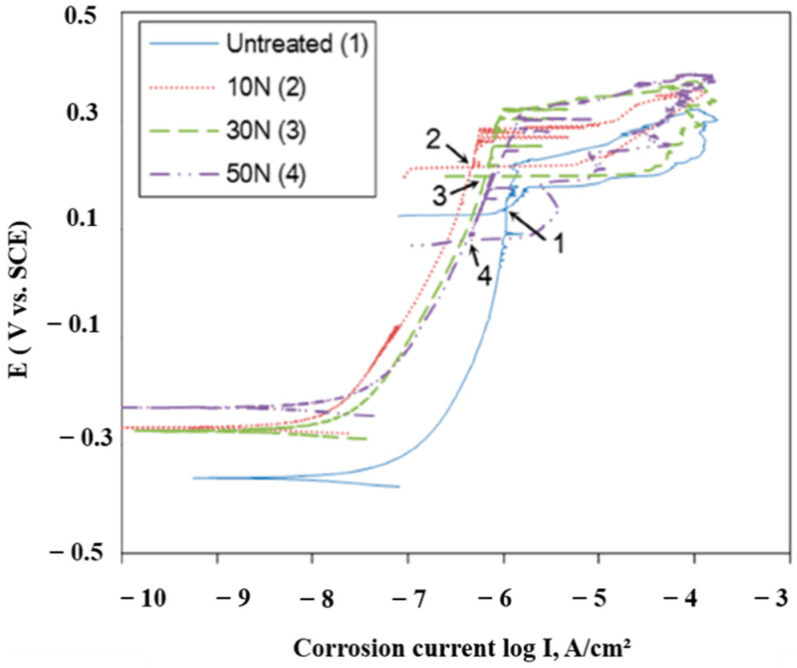
The cyclic potentiodynamic polarization curves obtained from USNM Ni 690 alloy subjected to 10 N, 30 N, and 50 N static loads. Reprinted with permission from ref. [112]. Copyright 2019, MDPI.

From an electrochemical perspective, the application of USNM can also improve the corrosion resistance of various alloys. For example, Kim and Kim [112] studied the effects of static load (spanning from 10 N, 30 N, and 50 N) on the pitting corrosion resistance of face-centered cubic (FCC) Ni 690 alloy in 1% NaCl solution. In this work, the WC tip (of 2.38 mm) was subjected to a reciprocating amplitude of 30 μm along a 0.07 mm pitch. According to their findings, the pitting potential (E_pit_) of each proceeding specimen (from untreated to 50 N) was markedly improved. Similarly, all the processed specimens demonstrated decreased passive current density (i_pass_), which acts as an indicator for a decreased corrosion rate (i_corr_), as per the widely known Faraday’s law [113]. However, the protection potential (E_protection_) decreased at the highest applied load, suggesting that the severe plastic deformation of 50 N might be detrimental to the electrochemical characteristics of the specimens. For a clearer insight into these findings, the corresponding cyclic potentiodynamic polarization (CPP) curves concerning these findings are shown in Figure 16.

Similar trends were also observed with the Nyquist plots derived from electrochemical impedance spectroscopy (EIS), where the radius of the capacitive curves was greater, indicating a greater polarization resistance. From a mechanistic perspective, the grain refinement from the USNM process predominantly influenced the corrosion behavior of this alloy. In fact, due to the increase in grain boundaries (from the refined microstructure), the chemical activity of the surface is largely improved, which helps to assist the sturdiness and reliability of the passive film. This implies that the amount of electron activity and diffusion increases, acting as central sites of oxide film nucleation. From another perspective, the increased rates of electron diffusion also enable a greater presence of triple-junctions, which can help increase the activity of neighboring electrons, thus improving the integrity of the passive film. The authors further supported these findings by calculating the lattice plane spacing of each specimen, as the XRD findings imply the formation of an amorphous state, which is quite reflective of the grain-refining characteristics of UNSM. Nonetheless, the inter-atomic spacing decreased, which further supports the findings.

It should be mentioned that the only problematic surface defect found in this work pertained to the wave formation topography formed along the overlapped processed regions, which act as sensitive sites for pitting initiation. Although the authors do not elucidate the influence of the roughness parameters on these findings, it can be assumed that increased roughness (as well as an increase in *R_sk_* and *R_ku_* values) increases the contacted area of the electrolytic solution of the severely deformed surface, which can exacerbate localized corrosion rates [114]. This would assume that the surfaces reflect the behavior of Wenzel’s model, which states that contacting liquid will penetrate the valleys of rough surfaces due to the strong adhesive forces [115]. Although this observation is far from the scope of this work, the authors suggest that future researchers investigate these characteristics and provide further insights into the influence of surface quality on the corrosion resistance of UNSM-based materials.

**Figure 17:**
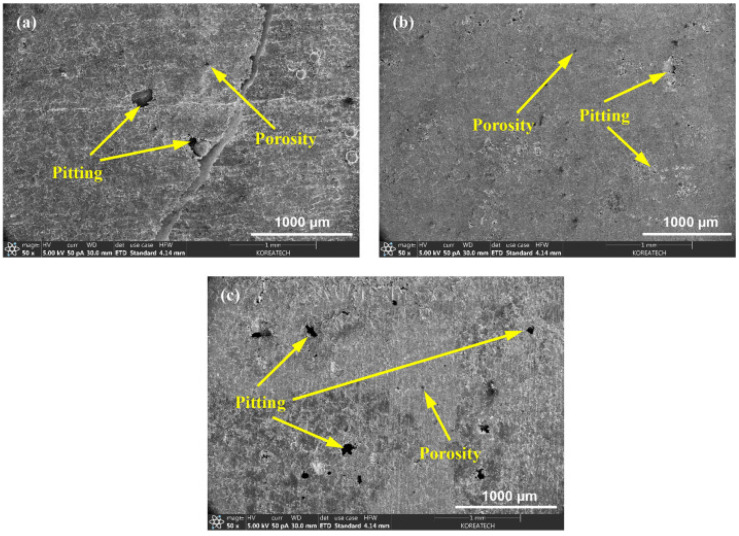
The pitting corrosion characteristics of (**a**) SLM 316L SS, (**b**) SLM 316L SS + UNSM, and (**c**) SLM 316L SS + UNSM + HT. Reprinted with permission from ref. [116]. Copyright 2020, Elsevier.

From an AM perspective, the application of UNSM can also greatly improve the corrosion resistance of 316L coatings fabricated by selective laser melting (SLM), as shown by Amanov [116]. This work applied UNSM at R and at HT. For reference, these samples are referred to as UNSM + RT and UNSM + HT. The UNSM processing parameters were set to a normal load of 30 N, an amplitude of 30 μm, a frequency of 20 kHz, and a moving speed of 2000 mm/min. Understanding that AM components tend to suffer from various surface defects, the primary focus of this work was to study how temperature (during UNSM) can control the surface quality and porosity while maximizing corrosion resistance in a 3.5% NaCl solution. Interestingly enough, the authors found that although the UNSM + RT demonstrated a lesser i_corr_ value than the as-printed substrate, applying heat treatment during UNSM decreased the corrosion resistance. Namely, the degree of localized pitting regions resulted in increased corrosion rates, as is evident from the combination of the decreased corrosion potential (E_corr_) and positive shifting of the Tafel curves. A visualization of the chloride-attacked regions is shown in Figure 17.

Although Amanov [116] did analyze these observations, the decrease in corrosion resistance for the UNSM + HT substrate can also be attributed to the change in surface roughness from the process. As indicated by his findings, the surface roughness of the UNSM + RT and UNSM + HT substrates is quite similar; however, the standard deviation of the UNSM + HT substrate is much greater. Although other surface roughness parameters were not studied, this finding most likely represents that the influence of heat treatment resulted in diffusion, which would increase the degree of plastic deformation along the surface during UNSM. Consequentially, the surface would have a greater deviation of peaks and valleys, acting as preferential sites for pitting corrosion. This aspect, combined with the less refined microstructure, most likely prevented the proper formation of the passive film along the surface and increased the likelihood of pitting corrosion along the surface.

In understanding the key mechanisms of the corrosion resistance of UNSM in chloride-based solutions, many have also studied its corrosion resistance in other solutions such as stagnant simulated body fluid (SBF) for biomedical applications. For example, Hou et al. [57] studied the effect of UNSM on AZ31B Mg alloy for orthopedic applications. In this work, UNSM was applied using a normal load of 5 N, a vibrational frequency of 20 kHz, a scanning speed of 1000 mm/min, a vibratory amplitude of 8 μm, and an interval of 0.01 mm. It was found that although the UNSM substrate demonstrated a nobler E_corr_ value, i_corr_ was found to be increased. This finding was largely attributed to the change in surface roughness as micro-galvanic coupling from both the anodic and cathodic regions accelerated the corrosion rate during Mg dissolution, which can be described by the following corrosion reaction.

(6) $$\begin{array}{c} \mathrm{Mg}+2H_{2}O\to \mathrm{Mg}{\left(\mathrm{OH}\right)}_{2}+H_{2}\;\left(\mathrm{gas}\right) \end{array}$$

Given that this reaction is accelerated, this can cause quite negative implications in an implant operation as localized reactions can result in increased toxins, reducing the usefulness of the implant. Consequentially, this will cause the host body to reject the implant altogether. Although SPD techniques refine the dislocation density of Mg alloys, which helps to form a robust and stable film, the increase in surface roughness results in an increased exposed area during electrochemical testing. Due to the lack of a low surface finish, the stability of the film generated during corrosion testing can fluctuate, which can cause metastable-like behaviors and thus increase the likelihood of accelerated corrosion rates. This was evident in this work as the P and Ca relation ions had a greater tendency to attack the surface of the UNSM substrate, as reflected by the Tafel curves. To mitigate these defects, the authors suggest that a separate post-processing technique be used to smoothen the surface, thus reducing the likelihood of enhanced corrosion rates.

## 5. Recent Advances of UNSM

**Figure 18:**
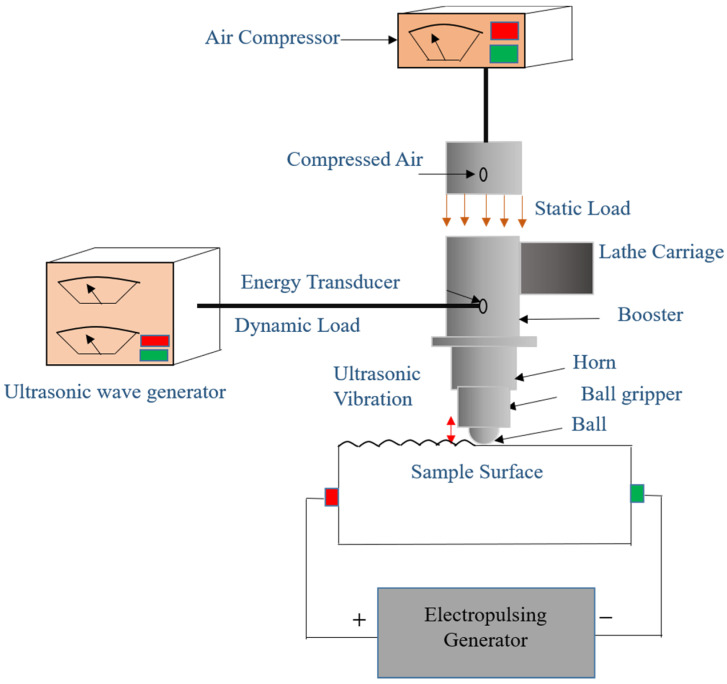
Schematic of EP-UNSM.

To enhance the existing benefits of UNSM, scholars introduced modified USNM techniques such as continuous current-assisted UNSM (CC-UNSM), electro pulsing-assisted USNM (EP-UNSM), and laser-assisted UNSM (LA-UNSM). These techniques can significantly enhance the surface mechanical properties and surface integrity compared to conventional UNSM. Researchers have demonstrated that pulsed current assisted with surface modification techniques could enhance the effectiveness of the process [117,118]. The conventional UNSM technique integrated with electropulsing led to the development of EP-UNSM. The electric pulsing during the process improves the plasticity of the processed material. Compared to CC-UNSM, EP-UNSM is more effective and most commonly used for developing deeper SPD’ed and S^2^PD’ed layers [119,120]. This is attributed to the higher peak current during electropulsing than continuous current for the same current density. In addition to that, electropulsing causes thermal and athermal effects, promoting dislocation mobility and atom diffusion [121]. During the EP-UNSM process, a high electropulsing current is applied to the substrate for a short period. This causes a reduction in the flow stress of the material and improves the substrate’s plasticity. The schematic of the EP-UNSM technique is shown in Figure 18.

Ye et al. [122] studied the effect of EP-UNSM on the mechanical properties and microstructural evolution of Ti-6Al-4V. The reported surface hardness for the base material was 315 Hv, which increased to 364 Hv after the UNSM and a 300 µm thick SPD layer was observed. The EP-UNSM treatment increased the hardness to 407 Hv and the SPD layer thickness to 550 µm. The authors reported that the surface roughness of the substrate was 0.918 µm, which was reduced to 0.028 µm by conventional UNSM, which further reduced to 0.19 µm after EP-UNSM. The electropulsing and UNSM effect synergistically caused superior enhancement in the surface properties compared to USNM. The authors also reported a significant enhancement in wear resistance during UNSM compared to EP-UNSM. Ma et al. [123] demonstrated for the first time that EP-UNSM can enhance the plasticity of bulk metallic glass. The synergistic effect of electropulsing and UNSM generate a hybrid structure with the nanocrystals uniformly embedded in the amorphous matrix. The authors revealed an enhancement in plasticity from 0% to 2.03 ± 0.29%. The authors summarized that EP-UNSM is a promising technique to develop bulk metallic glasses with superior strength and plasticity.

**Figure 19:**
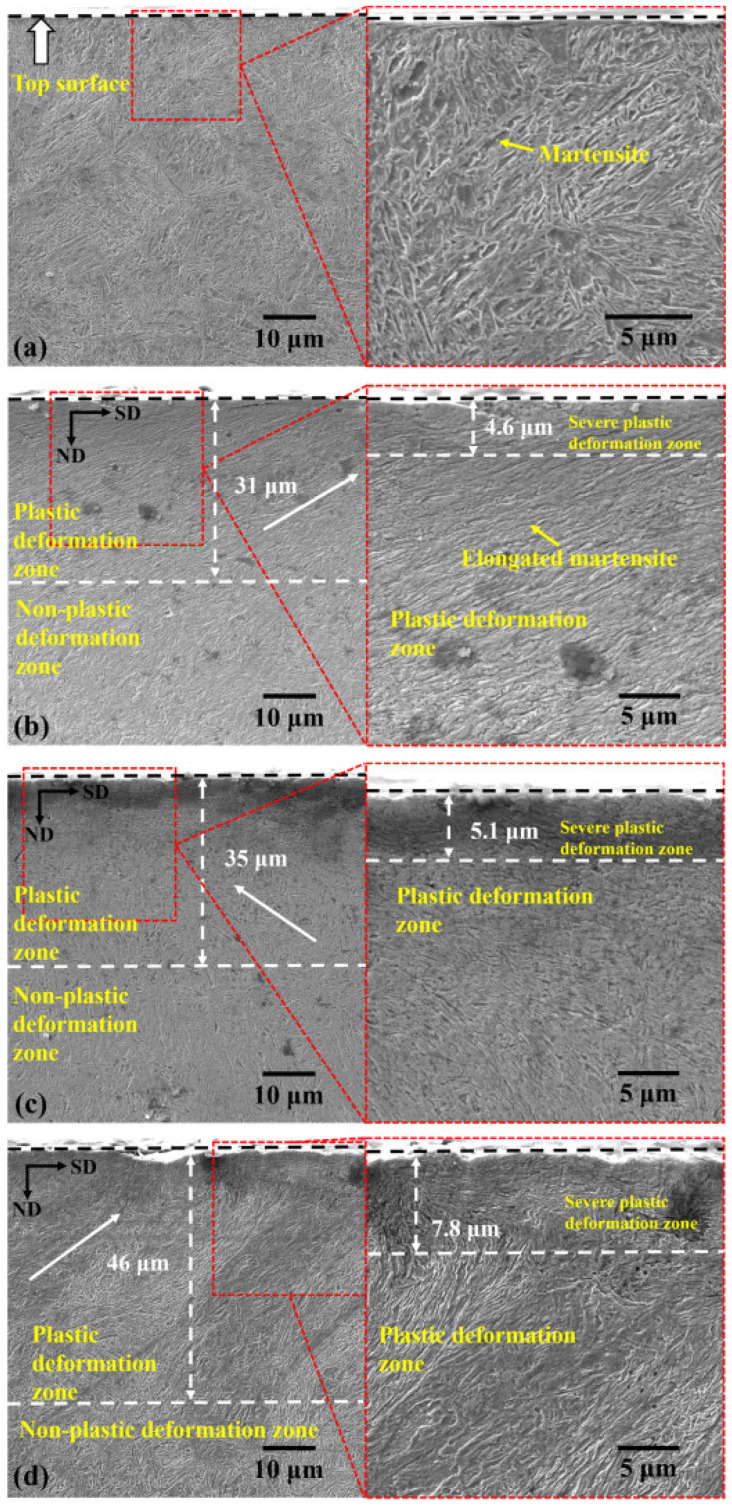
SEM of (**a**) 300 M steel, (**b**) UNSM’ed substrate, (**c**) CC-UNSM’ed substrate, and (**d**) EP-UNSME’ed substrate. Reprinted with permission from ref. [121]. Copyright 2020, Elsevier.

Zhao et al. [121] studied the effect of EP-UNSM on the microstructural features and mechanical properties of 300 M steel. In their experiments, the authors considered four specimens. The substrate, UNSM’ed substrate, CC-UNSM’ed substrate, and EP-UNSME’ed substrate. The authors revealed that the pulsation of current significantly enhanced the plasticity of 300 M steel compared to the continuous current UNSM and the conventional UNSM. The substrate microstructure consisted of tempered martensite. The authors observed an SPD layer of 31 µm on the surface of 300 M steel. In this SPD’ed layer, up to 4.6 µm from the surface S^2^PD’ed regions were reported. In addition, in the S^2^PD’ed region, refined lath martensite was observed, which lies in a direction parallel to the UNSM’ed direction. The micrographs of 300 M steel, UNSM’ed substrate, CC-UNSM’ed substrate, and EP-UNSME’ed substrate are shown in Figure 19.

During CC-UNSM, the depth of the SPD layer increased to 35 µm, and the S^2^PD region thickness increased to 5.1 µm. The enhanced SPD layer and S^2^PD region thickness is attributed to the improved plastic deformation ability of the specimen due to the rise in temperature during the CC-UNSM process. A significant refinement of lath martensite was observed. When EP-UNSM was adopted, the SPD layer thickness and the S^2^PD region thickness increased to 46 µm and 7.8 µm. The authors revealed that during CC-UNSM and EP-UNSM, the rise in temperature of the specimen was the same, and EP-UNSM was more efficient in generating deeper SPD layers and S^2^PD regions. The authors reported that EP-UNSM is a superior technique for developing a deeper SPD layer and S^2^PD region thickness, which can remarkably improve the surface properties.

Several studies demonstrate improved surface mechanical components by combining UNSM techniques with other surface modification techniques or using heat treatment methods [42,75,91,96,116]. Amanov and Pyun [91] conducted various tests to study the influence of local heat treatment (LHT) on Ti_6_Al_4_V alloy with and without the UNSM process. The results indicated an improvement in hardness with LHT alone. However, further enhancement was found on specimens subjected to combined UNSM and LHT processes. Even though the LHT followed by UNSM resulted in a significant improvement in hardness, it did not have any improvement in the wear resistance. Amanov [116] a similar study on SS 316L synthesized by selective laser melting (SLM). The surface roughness was decreased considerably after the UNSM treatment at HT. In addition, they achieved a lower COF and a slightly higher wear rate with the UNSM at HT compared to the UNSM at RT.

The property enhancement of the material’s surface by combining SP and UNSM is another interesting area where the material’s properties can be significantly improved. AISI 4340 alloy, having high strength and toughness, is a material that finds applications as aircraft landing gear and other structural applications. The enhancement of the surface properties of this alloy using SP and UNSM was explored by Karimbaev et al. [93]. The result showed improvement in fatigue characteristics with SP alone, which was further improved by combining SP and UNSM. Still, the highest fatigue properties were obtained with UNSM alone. Amanov et al. [42] explored the advantages of combining SP and UNSM in AISI 304 for fatigue properties. They validated that the combination of SP + UNSM achieved better properties than SP alone. The UNSM process displayed better fatigue properties compared to the combination of SP and UNSM. This is because of the high RCS induced by the UNSM process compared to the combination of SP and UNSM. Efe et al. [124] examined the improvement in microstructural and mechanical characteristics of AA7075 aluminum alloy by combining SSP and UNSM. The experiment followed two steps, SSP followed by UNSM and UNSM followed by SSP. The surface roughness reduction associated with UNSM was much more pronounced than SSP. UNSM, followed by SSP, was efficient in upgrading the frictional properties.

## 6. Applications of UNSM

UNSM is a promising and potential surface modification technique based on the mechanical impact that can significantly enhance the surface properties. Furthermore, the beneficial nature of UNSM led scholars to adopt UNSM in diverse fields of application to enhance the performance and longevity of engineering components. The application of UNSM is not limited to any particular area. Considered in a broader sense, the application of UNSM means the enhancement of the surface properties of the already available materials or the performance as a post-processing technique in improving the properties achieved by other manufacturing techniques.

One of the main areas of UNSM application is in the post-processing of AM’ed components. The unique properties of 3D-printed metals find potential applications in the aerospace and biomedical industries. However, the poor surface finish, high tensile residual strength, and high surface porosity of these materials lead to inferior mechanical properties. The capabilities of UNSM to improve surface properties and induce RCS on the surface of materials promoted UNSM as an effective post-processing technique in enhancing the capabilities of AM components.

Zhang et al. [90] studied the effect of UNSM on the fatigue properties of 3D-printed titanium alloys. They observed that the UNSM treatment improved the surface finish, induced a high RCS, and reduced subsurface porosity, substantially enhancing the rotation bending fatigue properties. Similarly, Ma et al. [61] explored the use of UNSM as a post-processing technique on AM’ed Ni-Ti alloys. Owing to the shape memory properties and superelastic behavior, the AM’ed components of Ni-Ti alloys are finding potential applications in the biomedical industry. Still, there is some concern regarding the release of toxic Ni ions due to the poor surface finish and high surface porosity of the AM’ed Ni-Ti alloys. This drawback is significantly reduced by the simultaneous ultrasonic striking and burnishing of the UNSM process on the AM’ed components. The resulting component has a better surface finish, lower subsurface porosity, and higher resistance to wear and corrosion. Amanov [105] investigated the effect of UNSM as a post-processing technique on AM’ed Co-Cr-Mo and studied the tribological and tribocorrosion properties. Co-Cr-Mo alloy finds application as a bearing surface for manufacturing the femoral head in metal-on-metal bearings. The UNSM process on the alloy was carried out at RT and HT. The UNSM-treated sample at HT showed better tribological and tribocorrosion properties than the AT-treated and as-printed alloy samples. The enhancement of the tribocorrosion resistance (TCR) of the AM’ed Co-Cr-Mo alloy, which is post-processed by UNSM, has several biomedical applications. The improvement of the surface properties of the UNSM-treated AM’ed aluminum alloy is examined by Ma et al. [74]. The UNSM process reduced the surface roughness (Ra) of the studied Al alloy from 18 μm to 3.5 μm, showing the potential of UNSM as a post-processing technique of AM’ed components.

The aerospace and automotive industry is another area where the capabilities of UNSM can be effectively implemented. Kattoura et al. [124] investigated the use of UNSM in improving the fatigue life of ATI 718 plus. 718 plus is a Ni-based superalloy having a maximum usage temperature of 650 °C. Due to the superior mechanical properties of ATI 718 plus alloys over other conventional Ni-based superalloys, the 718 Plus alloys are starting to find wide applications in the aerospace industry. The UNSM processing on the 718 plus alloys showed improved fatigue life through the near-surface microstructural changes, high RCS, and surface hardening induced by UNSM. The effect of UNSM treatment prior to nitriding on the surface of 300 M steel is investigated by Zhao et al. [77]. The 300 M steel is a low-alloy, high-strength steel containing tempered martensitic phase. The high strength of this material often finds application as landing gear in aircraft. However, the inferior pitting corrosion resistance of 300 M steel in the chloride environment is a concern. To enhance the pitting resistance, the authors performed nitriding on UNSM-treated and untreated specimens. The researchers found significant improvement in surface hardness, wear resistance, and enhanced corrosion resistance in the UNSM-treated specimen than in the untreated specimen. Zou et al. [98] employed the UNSM treatment on DZ2 axle steel to improve the wear resistance and surface integrity. This steel is used for several structural applications and specifically for high-speed trains. The multiple UNSM treatments resulted in grain refinement in the near-surface of the DZ2 steel. There is an appreciable improvement in the RCS and surface microhardness for the UNSM-treated steel compared to untreated steel. This resulted in a significant improvement in wear resistance under sliding conditions.

High bio-corrosion resistance and biocompatibility are the fundamental characteristics required for alloys in implant applications. Recently, a novel Ti alloy, TNTZ (Ti-29, Nb-13, Ta-4.6, Zr) was introduced as a potential candidate for implant applications. Kheradmandfard et al. [48] applied UNSM treatment to the TNTZ to investigate the wear properties and bio-functionalities improvement. The UNSM-treated TNTZ showed almost seven times higher wear resistance than the untreated one. Furthermore, based on the cell culture test, there was a significant increase in the cell adhesion, spread, and proliferation of cells on the UNSM-treated samples, which indicated improved biocompatibility due to the UNSM treatment. Aranov et al. [125] performed UNSM on SS 316L for biomedical application as a coronary artery stent (CAS). CAS is a small metal tube, capable of expanding, which is implanted in the human body to keep the lumen open by which normal blood flow can be provided. This is being used extensively to open up occluded coronary arteries. Because of several advantageous properties (including biocompatibility and good mechanical properties), SS 316L is widely used for CAS applications in biomedical industries.

In summary, UNSM is a novel and promising SPD technique that can provide superior surface mechanical properties and surface integrity. The remarkable enhancement in surface properties is due to UNSM potentially enhancing the longevity of engineering materials used in the diverse field of application.

## 7. Conclusions

This review article elucidated a comprehensive discussion on the mechanical impact-based surface modification technique, namely UNSM. UNSM is a promising SPD method that remarkably enhances surface integrity and surface mechanical properties. This review exclusively focuses on the current state-of-the-art usage of UNSM on various engineering materials that are specifically used for diverse applications. Furthermore, this review explores the nanostructuring and formation of gradient nanostructured surface (GNS) layers during UNSM. The formation of GNS layers and the introduction of high-density dislocations, twinning, high-angle grain boundaries, subgrains, and grain-refinement mechanisms were explained. In addition, recent advancements of UNSM, such as EP-UNSM and a combination of UNSM with other surface modifications, were summarized. Finally, different applications of UNSM, such as the automotive, aerospace, nuclear, and chemical industries, were elucidated. UNSM has superior control over plastic deformation and surface roughness; hence, it can potentially prevent fatigue, corrosion, and wear failure. UNSM is a potential technique, compared to other SPD techniques, that can significantly impact materials used to manufacture mechanical components expected to operate in extreme and dynamic conditions. These advantages make UNSM an ideal candidate for industries compared to other SPD techniques. UNSM is highly recommended for obtaining remarkable surface mechanical properties and surface integrity on components for industrial applications and among scholars working in the surface modification of engineering materials. In the near future, a transition from other SPD techniques to USNM can be expected. Overall, this review can provide a deeper understanding of the effect of the UNSM technique on the mechanical properties and microstructural features of various engineering materials.

## Figures and Tables

**Table 1:** Mechanical properties and microstructural features of USNM-treated substrate materials.

Materials	Static Force	Roller Material, Hardness, and Diameter	Frequency and Amplitude	Observation
304 ASS [41]	20 N	WC; Not Specified	20 kHz; 10 µm	SPD layer of 50 µm thickness. High hardness of 7 GPa. Increase in yield strength by 85.29%. Fatigue life improved by 7.67 times Maximum RCS of 1400 MPa
SUS 304 [44]	70 N, 90 N, 110 N and 130 N	WC; Not Specified, Diameter 2.38 mm	~20 kHz; 30 µm	Surface roughness decreased from 1.05 µm to 0.32 µm. Maximum RCS of 43.1 MPa. Maximum fatigue strength of 510 MPa at 90 N, 82.14% increase.
AISI 304 [96]	Not Specified	WC; Not Specified	20000 or more per second; Not Specified	Surface roughness improved from 1.7 µm to 1.3 µm. Maximum RCS of 1100 MPa. Surface hardness increased from 220 Hv to 390 Hv
300 M [77]	50 N	WC; Not Specified	20 kHz; 24 µm	Surface roughness decreased by 40% compared to the nitrided specimen. Surface hardness increased by 40.64% compared to base material Surface hardness increased by 13.40% on the nitrided specimen.
A100 [86]	50 N	Cemented WC; Not Specified	20 kHz; 24 µm	Surface hardness increased from 574 Hv to 707 Hv (23.17% increase) Maximum RCS of 1706 MPa is induced, which is a 4.4 times increment. 14 times increase in plain fatigue life and 2 times increase in fretting fatigue life.
AISI 4340 [96]	40 N	WC; Not Specified; 2.38 mm	20 kHz; 30 µm	R_a_ and R_z_ decreased by 72.7% and 52.9%. Surface hardness increased by 38.59%. Maximum RCS of 717 MPa. Increase in fatigue strength by 45.45%. Increase in fatigue life of 214 times.
AA7075-T651 [51]	1 kg	WC; Not Specified	20 kHz; 8 µm	Fatigue life increased by 11 times when UNSM was applied on normal material. The fatigue life was 4 times higher in specimens subjected to corrosion for 2 h followed by UNSM than in specimens subjected to corrosion alone for 2 h Surface roughness increased from 2.3 µm to 2.4 µm for specimen subjected to corrosion for 2 h The maximum RCS was 600 MPa
AA7075-T6 [75]	30 N	WC; Not Specified; 2.38 mm	20 kHz; 30 µm	Surface roughness decreased by 15.75% Maximum RCS of 780 MPa. Microhardness increased by 26%. The wear rate improved by 2.75 times. COF was reduced by 6.4%.
CP Ti [47]	30 N	WC; Not Specified; 2.38 mm	20 kHz; 30 µm	Grain refined to 200 nm. Surface hardness increased by 32.19%. Maximum RCS of 1279.4 MPa
Ti64 [90]	60 N	WC; Not Specified; 2.38 mm	20 kHz; 30 µm	Grain refined to 1.2 µm and 0.8 µm for α and β. Surface hardness increased by 15.55%. Maximum RCS of 1142.7 MPa.
Ti-Nb-Ta-Zr [46]	25–40 N	WC; Not Specified	20 kHz; 24 µm; 40 µm	Decrease in surface roughness by 44.32% Surface hardness increased by 13.40%. Maximum RCS of 1094 MPa. Fatigue life increased by 100% in slow rotation and 160% in fast rotation during UNSM.
Inconel 718 [54]	40 N	WC; Not Specified; 2.38 mm	20 kHz; 40 µm	GNS layer formed up to a depth of 60 nm–200 nm. Increase in hardness of 102.63%.
ATI 718 plus [69]	10 N/50 N	WC; Not Specified; 2.5 mm	25 kHz; 40 µm	Grain refined up to 21.95 nm. Increase in surface hardness by 44%. Maximum RCS was higher than 1000 MPa. Fatigue life improved by 5.25 times
Inconel 690 [93]	20–40 N	WC; Not Specified; 2.5 mm	25 kHz; 8–16 µm	Surface hardness increased by 44.1%. Maximum RCS of 1376 MPa. Yield strength increased by 13%. Endurance strength increased by 13.5%.
CP Cu [104]	50 N	WC; Not Specified; 2.38 mm	20 kHz; 50 µm	Grain refinement up to 200 nm. Surface hardness increased by 142.65%. Maximum RCS of 1.5 GPa. Yield strength enhanced by 44.15%. Tensile strength increased by 6.75%.
AZ31B Mg Alloy [57]	5 N	WC; Not Specified; 4 mm	20 kHz; 8 µm	Surface roughness reduced by 21.19% Surface hardness increased by 63.98%. Yield strength increased by 43.48%.
AZ91D Mg Alloy [56]	10 N; 20 N; 30 N	WC; Si_3_N_4_; 2.38 mm	20 kHz; 30 µm	Grain refinement up to 39 nm. Surface hardness increased by 28.26%. The wear rate was reduced by 30%. COF was reduced by 23%.
Co-Cr-Mo Alloy [105]	50 N	WC; Not Specified; 2.38 mm	20 kHz; 30 µm	Surface roughness was reduced by 83.02% and 87.69% at room temperature (RT) and high temperature (HT) Surface hardness increased by 35.1% and 44.3% at RT and HT. Yield strength increased by 3.4% and 11.78% at RT and HT. Tensile strength increased by 5.5% and 10.8% at RT and HT. COF reduced at 23.36% and 48.07%. Specific wear rate (SWR) reduced by 43.1% at RT and 77.3% at HT.
CoCrFeMnNi High Entropy Alloy [63]	10 N; 20 N; 60 N	WC; Not Specified; 2.4 mm	20 kHz; 30 µm	Increase in surface hardness by 98.28%. Yield strength increased by 142.49%.
Nickel-Titanium Shape Memory Alloy [61]	3 kg	WC; Not Specified; 2.4 mm	20 kHz; 20 µm	Surface roughness decreased from 12.1 µm to 9.0 µm. Surface hardness increased from 304 Hv to 408 Hv (34.2% increase). Corrosion current decreased from 157 nA to 53.1 nA.
Nickel-Titanium Shape Memory Alloy [106]	20 N	WC; Not Specified	20 kHz; 12 µm	Corrosion resistance improved Surface hardness increased by 21.81%. Cell adhesion increased

## Data Availability

Data sharing is not applicable to this article.

## Abbreviations

AM	Additive Manufacturing
EP-USRP	Electropulsing-Assisted Ultrasonic Surface Rolling Process
GNS	Gradient Nanostrcutured Surface
HVOF	High-Velocity Oxygen Fuel
LSP	Laser Shock Peening
LSSP	Laser Shock Surface Patterning
RCS	Residual Compressive Stress
SP	Shot Peening
SPD	Severe Plastic Deformation
SSP	Severe Shot Peening
SS	Stainless Steel
SEM	Scanning Electron Microscope
SMAT	Surface Mechanical Attrition Treatment
SBF	Simulated Body Fluid
SFE	Stacking Fault Energy
TEM	Transmission Electron Microscopy
USP	Ultrasonic Shot Peening
UIP	Ultrasonic Impact Peening
USRP	Ultrasonic Surface Rolling Process
UNSM	Ultrasonic Nanocrystal Surface Modification
